# No superior surgical treatment for secondary osteochondral defects of the talus

**DOI:** 10.1007/s00167-017-4629-0

**Published:** 2017-07-07

**Authors:** Kaj T. A. Lambers, Jari Dahmen, Mikel L. Reilingh, Christiaan J. A. van Bergen, Sjoerd A. S. Stufkens, Gino M. M. J. Kerkhoffs

**Affiliations:** 1 Department of Orthopedic Surgery, Academic Medical Center, University of Amsterdam, Meibergdreef 9, 1105 AZ Amsterdam, The Netherlands; 2 Academic Center for Evidence Based Sports Medicine (ACES), Amsterdam, The Netherlands; 3 Amsterdam Collaboration for Health and Safety in Sports (ACHSS), AMC/VUmc IOC Research Center, Amsterdam, The Netherlands; 4 Department of Orthopedic Surgery, Amphia Hospital, Breda, The Netherlands

**Keywords:** Osteochondral defects, Talus, Failed primary surgery, Surgical treatment, Success rate

## Abstract

**Purpose:**

The purpose of this systematic review was to identify the most effective surgical treatment for talar osteochondral defects after failed primary surgery.

**Methods:**

A literature search was conducted to find studies published from January 1996 till July 2016 using PubMed (MEDLINE), EMBASE, CDSR, DARE and CENTRAL. Two authors screened the search results separately and conducted quality assessment independently using the Newcastle–Ottawa scale. Weighted success rates were calculated. Studies eligible for pooling were combined.

**Results:**

Twenty-one studies with a total of 299 patients with 301 talar OCDs that failed primary surgery were investigated. Eight studies were retrospective case series, twelve were prospective case series and there was one randomized controlled trial. Calculated success percentages varied widely and ranged from 17 to 100%. Because of the low level of evidence and the scarce number of patients, no methodologically proper meta-analysis could be performed. A simplified pooling method resulted in a calculated mean success rate of 90% [CI 82–95%] for the osteochondral autograft transfer procedure, 65% [CI 46–81%] for mosaicplasty and 55% [CI 40–70%] for the osteochondral allograft transfer procedure. There was no significant difference between classic autologous chondrocyte implantation (success rate of 59% [CI 39–77%]) and matrix-associated chondrocyte implantation (success rate of 73% [CI 56–85%]).

**Conclusions:**

Multiple surgical treatments are used for talar OCDs after primary surgical failure. More invasive methods are administered in comparison with primary treatment. No methodologically proper meta-analysis could be performed because of the low level of evidence and the limited number of patients. It is therefore inappropriate to draw firm conclusions from the collected results. Besides an expected difference in outcome between the autograft transfer procedure and the more extensive procedures of mosaicplasty and the use of an allograft, neither a clear nor a significant difference between treatment options could be demonstrated. The need for sufficiently powered prospective investigations in a randomized comparative clinical setting remains high. This present systematic review can be used in order to inform patients about expected outcome of the different treatment methods used after failed primary surgery.

**Level of evidence:**

IV.

## Introduction

For the treatment of talar osteochondral defects (OCDs), a wide variety of treatment strategies have been reported in the literature. These strategies can be divided into a number of treatment groups: conservative treatment, bone marrow stimulation (BMS), retrograde drilling, osteo(chondral) transplantation, cartilage implantation and chondrogenesis-inducing therapies (CIT). In general, the different surgical treatments of talar osteochondral defects have good results, but although the great majority of defects improve after surgical treatment, a minority of lesions will fail first-line surgical treatment and therefore remain symptomatic [30, 40, 63]. This was exemplified by studies conducted by Yoon et al. [62] and Choi et al. [11], in which 11% of 399 patients and 6.7% out of 120 ankles, respectively, required revision surgery. These numbers indicate that secondary and tertiary surgical treatment for talar OCDs is not uncommon [54].

Although these patients generally represent a therapeutic challenge to the orthopaedic surgeon, most research in the past decades has focused on the treatment of primary talar OCDs. To a lesser extent, researchers in the orthopaedic field have attempted to identify promising surgical treatment options for non-primary lesions [62]. Systematic reviews aspiring to determine the most effective treatment option for talar OCDs so far include patient populations of both primary and non-primary lesions [30, 40, 63]. To our knowledge, no previously published systematic review has exclusively investigated the clinical effectiveness of different surgical treatment options for talar OCDs that have failed primary surgery. Hence, the aim of the present systematic review is to identify the most effective surgical treatment for talar OCDs after failed primary surgery.

## Materials and methods

### Search strategy

The systematic review was prospectively registered at the PROSPERO register [10].

Electronic databases PubMed (MEDLINE), EMBASE, CDSR, DARE and CENTRAL were screened from January 1996 till July 2016 for potential articles of interest (Appendix 1). Since not all titles or abstracts in these databases clearly describe whether the OCD surgery was primary or secondary, the authors deliberately did not use narrower terms such as “secondary”, “tertiary” or “failed primary” in our search as this would potentially exclude eligible studies. In addition to this, a backward citation chaining strategy was used by scanning the literature lists of suitable studies.

### Eligibility criteria and study selection

All studies that assessed the effectiveness of different surgical treatment strategies for previously failed surgical intervention of talar OCDs were included in the systematic review. The criteria for exclusion were primary OCDs, a study cohort of <5 patients, aged <16 (the age around which epiphyseal fusing takes place), concomitant distal tibial lesions and a follow-up of less than 6 months. When necessary, authors were contacted via email to provide separate data for those patients with non-primary lesions only and/or for patients ≥16 years old and/or to exclude concomitant tibial lesions. When no reply was received from an author, contact was attempted once more by a second email. In the cases of further lack of response a third, or when necessary a fourth email was sent. Ultimately, if no response was recorded from the corresponding author, then their specific article was excluded. Independent evaluation of the articles was conducted and then discussed by two reviewers (K.L. and J.D.). In cases of disagreement between the reviewers, the opinion from an independent third investigator (G.K.) was found to be decisive. Studies were not blinded for author, affiliation or source, and no limitations were put on language and publication status of screened articles.

### Quality assessment

To assess the quality of the included studies, a Newcastle–Ottawa scale (NOS) modified for talar OCD was used [58]. This scale assesses the methodological quality of non-randomized studies (Appendix 2). Each included study was graded on methodological quality by two independent reviewers (K.L. and J.D.).

### Data extraction

The following study and patient characteristics were retrieved: age, gender, number of patients and ankles, symptom duration, location, side, size and stage of the defect, type of surgery, clinical scoring system utilized, history of ankle trauma reported, follow-up duration and the OCD classification staging system reported. Pre-operative and post-operative clinical outcome scores were extracted. Clinical values of the last recorded follow-up were used. The treatment strategy in question was defined to be successful when a good or excellent result was reported at follow-up in combination with an accepted scoring system such as the AOFAS (American Orthopaedic Foot and Ankle Society) Ankle/Hindfoot scale, the FAOS (Foot and Ankle Outcome Score) or the Hannover Scoring System. For clarification, an ankle was considered to be successfully treated when at the last follow-up there was a post-operative AOFAS score of 80 or above [27]. When the FAOS (Foot and Ankle Outcome Score) was used, a score of also 80 or above was regarded to be a successful treatment [50]. When the original ankle score by Mazur et al. [35] was used, a score of 70 or higher was considered to be a successful treatment. In one case, a modified Mazur score was used, which had a 100-point scale. In this case, a score of 80 or higher was considered successful treatment [26].

### Statistical analysis

Since a formal meta-regression was not methodologically possible for the included studies (i.e. studies were included with highly different methodological study types and small numbers), it was decided to present the results per study by means of a forest plot. A simple pooling method was used to combine data from different studies that had similar methodologies. 95% binomial proportion confidence intervals were calculated for the success percentages of each study with the Wilson score interval and included in the forest plot (*CIA, Confidence Interval Analysis for Windows, version 2.2.0*) [8]. To compare between groups with categorical variables, a Fisher’s exact test was used (*SPSS version 23.0, IBM Corp*.). All reported p values are two-sided, and statistical significance was set at *p* < 0.05.

## Results

### Search results

**Fig. 1:**
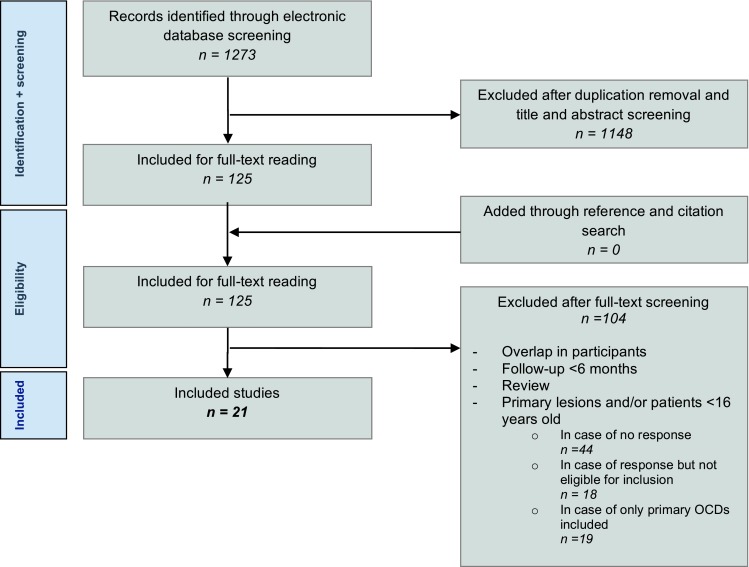
Literature selection algorithms—Preferred Reporting Items for Systematic Reviews and Meta-Analyses (PRISMA)

The literature search in the selected databases yielded 1273 articles. After the application of the eligibility criteria to the titles and abstracts, potentially suitable articles were included for full-text review while ineligible articles were deleted. Subsequently, full-text articles were obtained and the eligibility criteria were applied again. After screening and discussion between the first two authors, there was overall consensus in most cases except for two where disagreement persisted, these were resolved via discussion with the senior author. In the end, a total of 21 studies were included (Fig. 1).

### Characteristics of included studies (Tables 1, 2, 3)

A total of 299 patients with 301 talar OCDs who failed primary surgery were included. The average age of the patients was 35 (range 18–57), and the percentage of females and males in our study sample was 35 and 65%, respectively. The right ankle was involved in 47% of the cases and the left ankle in 53%. The percentages of medial, lateral, central and combined medial and lateral location involvement were 64, 19, 15 and 2%, respectively. Size of the lesion or surface diameter was rarely described or impossible to extract and was therefore not incorporated into our review. The most frequently used clinical scoring system and osteochondral damage classification system were the American Orthopaedic Foot and Ankle Society Score (AOFAS) and the Berndt and Harty/Loomer Classification system [6, 27]. In total, 17 different scoring systems were found [3, 9, 13, 14, 21, 25, 27, 33, 38, 42, 50, 56, 57, 61] and ten different osteochondral damage classification systems [2, 6, 7, 15, 23, 31, 32, 36, 43, 48] were used (Tables 2, 3). It was only possible to extract Berndt and Harty stages for 80 ankles: there were 0 (0%), 9 (11%), 21 (26%), 26 (33%) and 24 (30%) Berndt and Harty/Loomer [6] stage I, II, III, IV and V cases, respectively. In 87 of the 299 patients, a history of ankle trauma was reported. If divided through the number of treated ankles in the articles that report on history of trauma, this corresponds to 76.3%. The mean follow-up was 40 months [range 12–66].

**Table 1:** Demographics

Number of patients	299
Number of described OCDs	301
Mean age (range)	35 (18–57)
% Right/left	47/53
% Male/female	65/35
% Location (medial/lateral/central/combined)	65/19/15/2
% With history of trauma	76
Mean follow-up (range)	40 (12–66)

All described calculations are weighted

**Table 2:** Clinical scoring systems utilized for treatment of talar OCDs

Clinical scoring system	No. of studies
American Orthopaedic Foot and Ankle Society score (AOFAS)	17
Visual analogue scale (VAS)	7
Hannover Scoring System	3
Modified ankle grading system of Mazur, Schwartz and Simon	3
Short-Form-36	3
Foot and Ankle Outcome Score (FAOS)	2
Patient-based satisfaction score (developed by authors)	2
Short-Form-12	1
Numeric Rating Scale (NRS)	1
Foot Function Index (FFI)	1
Modified Cincinnati rating scale score	1
EQ-5D	1
Olgilvie–Harris score	1
Ankle Activity score (AAS)	1
AAOS Foot and Ankle Module Score	1
Ankle Osteoarthritis Scale (AOS) score	1
Subjective Ankle Hindfoot Score (AHS)	1

Some studies utilized >1 scoring system

**Table 3:** Systems utilized for osteochondral staging

Bone/cartilage classification systems	No. of studies
Berndt and Harty Classification System	8
International Cartilage Repair Society (ICRS)	3
Outerbridge classification	2
MOCART	2
Revised classification based on MRI appearances by Hepple et al.	1
Cheng–Ferkel grading system	1
International Cartilage Repair Society II (ICRS II)	1
Anderson’s modified MRI-based classification system	1
Mintz staging system	1
OsScore	1

Some studies utilized >1 classification system, and others did not utilize or describe a classification system

### Methodological quality

Of the 21 studies included, eight were retrospective case series, twelve were prospective case series and there was one randomized controlled trial (RCT). Full consensus was reached between the reviewers regarding the grading of methodological quality. Seventeen studies described that their research was conducted according to protocol. In fourteen studies, the described cohort was truly or somewhat representative to a talar OCD patient sample/population; in the other seven studies the group was either selected by an orthopaedic surgeon or there was no description of the cohort. In all studies, outcome was assessed through independent blind assessment or record linkage. In nine articles, the follow-up was complete or the loss to follow-up was reported to be smaller than 5%. In the other twelve articles, either the percentage loss to follow-up was more than 5% or the follow-up rate was not stated.

### Treatment strategies

**Fig. 2:**
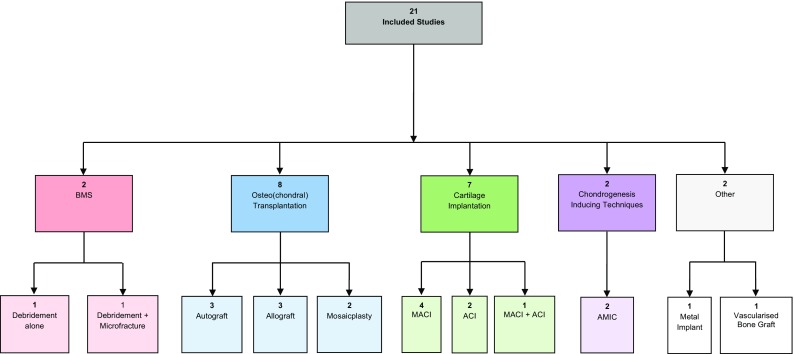
Flow chart of study inclusion and different treatment groups (*ACI* autologous chondrocyte implantation, *AMIC* autologous-matrix-induced chondrogenesis, *BMS* bone marrow stimulation, *MACI* matrix-associated chondrocyte implantation)

**Fig. 3:**
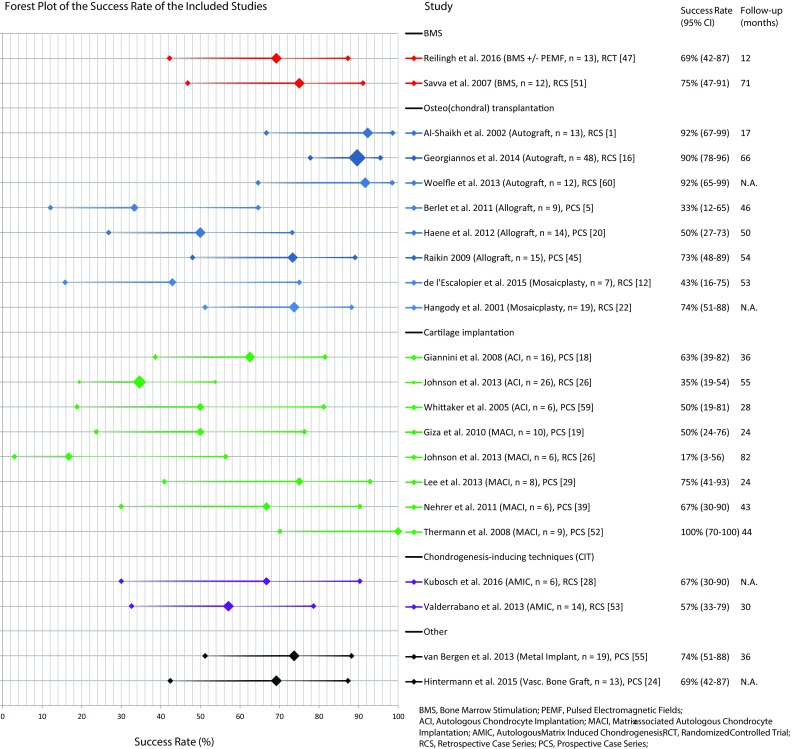
Forest plot of all included studies with the success rates per separate study sorted on treatment strategy and alphabetical order accompanied by number of ankles and follow-up duration (the size of the diamond representing the success rate is categorically adjusted for number of ankles included in the publications)

**Fig. 4:**
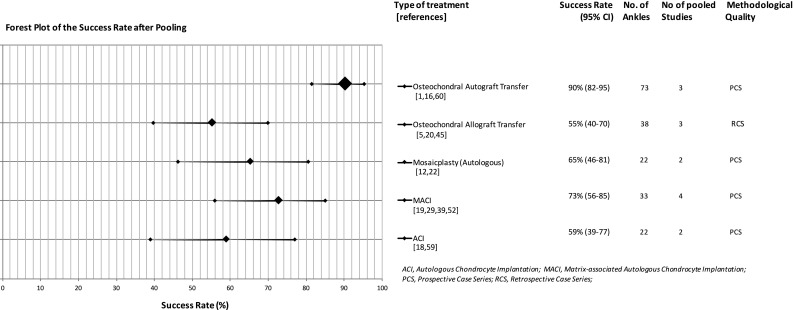
Forest plot of the success rates after pooling sorted on treatment strategy accompanied by the number of included studies, the success rate with confidence interval and the total number of ankles per group (the size of the diamond representing the success rate is categorically adjusted for the number of ankles included)

The treatment strategies were divided into the four previously indicated treatment groups: bone marrow stimulation (debridement and/or drilling), osteo(chondral) transplantation (autograft transfer, allograft transfer and mosaicplasty), cartilage implantation (MACI and ACI) and chondrogenesis-inducing techniques (AMIC). Two studies did not correspond to any of these groups and are presented separately (Fig. 2). All calculated success percentages with their respective confidence intervals are also visually presented in a forest plot (Fig. 3). The calculated success percentages of the pooled groups are shown in an additional forest plot (Fig. 4).

### Bone marrow stimulation (BMS)

Two studies described the outcome of BMS as a secondary treatment for a total of 25 treated ankles [47, 51]. One of these studies (Savva et al.) solely reported the results of repeat arthroscopic treatment [51]. The authors used the Berndt and Harty classification to grade the OCDs, but excluded cystic lesions as their treatment type (debridement) had been demonstrated to have poor outcomes in these type of lesions [49]. The second study by Reilingh et al. is an RCT investigating the effects of pulsed electromagnetic fields (PEMF) on debridement and microfracture [47]. Due to the fact that the authors indicated that there was no significant difference between the randomized treatment groups, patients from both groups were included. In this study, cystic lesions were not excluded. The two studies describe a post-operative AOFAS of 81 and 80. Success percentages of 75% (debridement alone) and 69% (debridement and microfracture) were calculated with confidence intervals of 47–91% and 42–87%, respectively.

### Osteo(chondral) transplantation

Osteochondral transplantation was the most commonly described procedure after failed primary surgery as eight studies were identified describing 135 patients corresponding to 137 treated ankles [1, 5, 12, 16, 20, 22, 45, 60]. Five of these studies were retrospective in design, and the other three were prospectively described case series. The mean follow-up for these studies was calculated to be 4 years (range 17 months–5.5 years). The included studies described different osteo(chondral) transplantation techniques. Three articles investigated a procedure with a classic osteochondral autograft transfer system (OATS) [1, 16, 60]. Two studies described a (autogenous) mosaicplasty procedure, which is similar to an OATS technique but uses multiple grafts creating a mosaic pattern [12, 22]. Three other studies described an allograft procedure [5, 20, 45]. A calculated success rate for all osteochondral transplantation techniques combined is not possible since the study designs were not similar enough for all studies. However, coincidentally all studies describing the allograft procedure were of the same level of evidence (retrospective case series), and all studies describing the autograft procedure and the two describing the mosaicplasty procedure were of the same level of evidence (prospective case series). It was therefore possible to use a simplified pooling method to combine data from these groups (Fig. 4). The autograft group after simple pooling consisted of 73 treated ankles and the allograft group of 38 treated ankles. This gave a calculated mean success rate for the autograft procedure of 90% [CI 82–95%] and a calculated mean success rate of 55% [CI 39.7–69.9%] for the allograft procedure. The success rate of the two studies describing a mosaicplasty procedure which was autogenous in nature for both studies was 65% [CI 46.2–80.6%]. A Fisher’s exact test showed no difference between mosaicplasty and the allograft transfer procedure (*p* = 0.449). A comparison between these two groups and the aforementioned autograft group was not performed since a mosaicplasty or an allograft procedure is often used for larger OCDs and a direct comparison would not be appropriate.

### Cartilage implantation

In total, seven suitable studies were found that described a form of cartilage implantation after failed primary surgery [18, 19, 26, 29, 39, 52, 59]. The two major groups were the classic autologous chondrocyte implantation (ACI) and the more recently developed matrix-associated chondrocyte implantation (MACI). In total, four studies described a MACI procedure [19, 29, 39, 52] and two described an ACI procedure [18, 59]. One study described both an ACI and a MACI procedure [26]. The latter was retrospective in design. All other studies in the cartilage implantation group were prospective, which allowed a simplified pooling method for both the MACI and ACI groups. Eighty-seven ankles were included in the cartilage implantation group as a whole and 39 of those received a MACI procedure and 48 received an ACI procedure. When combining data on the ankles from the studies methodologically suitable for pooling, thirty-three ankles in the MACI group and twenty-two in the ACI group were found. The calculated success rates were 72% [CI 56–85%] and 59% [CI 39–77%] for the MACI and ACI groups, respectively. A Fisher’s exact test showed no significant difference between these two groups.

### Chondrogenesis-inducing techniques (CIT)

Two studies that investigated the effects of a chondrogenesis-inducing technique were identified [28, 53]. Both are retrospective in design and performed an autologous-matrix-induced chondrogenesis (AMIC) procedure. The study by Kubosch et al. describes a procedure where the proximal tibia was used as a harvest site [28]. The study by Valderrabano et al. describes the iliac crest as a site to obtain the cancellous bone [53]. In total, 21 patients with 21 treated ankles were described in the two included studies. Both Kubosch and Valderrabano report high mean post-operative AOFAS scores of 91 and 86 points, respectively. The corresponding calculated success rates were 67% [CI 30–90.3%] and 57% [CI 32.6–78.6%], respectively. Both articles also included pre- and post-operative VAS scores for pain, which decreased from 8 and 5.5 to 1.7 and 2, respectively.

### Other

Two studies described a specific technique that could not be incorporated into any of the groups. The first article describes a novel metal implant in 20 patients after failed previous treatment [55]. Almost all lesions (18 out of 20) were classified as type V (cystic) lesions [6]. The defect was debrided after which the metal implant was introduced into the defect, and this occurred 2 years (mean) after the last failed surgical treatment. The median AOFAS score improved from 62 points pre-operatively to 87 points post-operatively. Of the nineteen patients, fourteen were successfully treated according to the AOFAS, resulting in a success percentage of 74% [CI 51–88%]. The second study included in this group was a transplantation method using a bone graft [24]. As the bone graft was vascularized, the authors decided not to include this study in the previously described osteochondral transplantation group. For the vascularized bone graft, they identified the medial condyle of the femur as an ideal site to harvest since it is sufficiently large and solid and has a contour similar to the talar surface. Data solely on the non-primary lesions were obtained, which included thirteen patients. The mean VAS for pain decreased from 5.8 to 1.7. The mean AOFAS hindfoot score increased from 66 points pre-operatively to 81 points post-operatively. Nine of the thirteen patients scored good to excellent according to the AOFAS which resulted in a success rate of 69% [CI 42–87%].

## Discussion

To the best of our knowledge, this is the first systematic review investigating the effectiveness of treatment options for talar osteochondral defects after failed primary surgical treatment. This is in contrast to previous reviews that focused either solely on primary or both primary and non-primary talar OCDs [30, 40, 63]. The differences in treatment approach for non-primary-treated OCDs often differ and are mainly based on expert opinion since evidence is limited. By means of the present review, it was the goal to give an evidence-based insight into the most effective surgical treatment option.

One of the major differences compared to studies focused solely on primary talar OCDs is that the most described treatment option is different. As for the healing of articular cartilage injuries, O’Driscoll postulated that the treatment options of these defects can be grouped according to four principles. It can be restored, replaced, relieved or resected [41]. It is generally expected that the first-line surgical treatment should at least incorporate restoration of the cartilage, which is normally accomplished by enhancing the intrinsic capacity of this cartilage and subchondral bone to heal itself. In a previous review on primary OCDs, BMS (including retrograde drilling) was the most described treatment method. Not unexpectedly, the most frequently described treatment method for secondary talar OCDs was not BMS but a type of replacement strategy.

In the literature, numerous treatment recommendations are given considering failed primary surgery, for example that patients with failures of previous arthroscopic treatment should be treated with osteochondral transplantation [17]. The recommendations are, however, not based on any concrete data. In this review, the success rates of BMS as a revision surgery were found to be 69 and 75%, which seems acceptable and lies within the success rate ranges found in primary OCD surgery. It, however, lies below the confidence interval after pooling of eleven retrospective studies describing primary surgery in 317 ankles which yielded an overall BMS success rate of 82% [CI 78–86%]. In the present review, only 25 ankles treated with BMS were evaluated. The calculated success rate is, however, still promising, and since BMS is a relatively non-invasive and inexpensive treatment, it should still be considered for certain patients with small secondary defects or at least be included in the shared decision process with the patient when discussing further treatment options after a previously failed primary operation [46]. It should also be noted that a study by Yoon et al. [62]—which was not included in the present review—described a clinically inferior outcome which corresponded to a calculated success rate of 32%. In this study, the authors compared repeat arthroscopy to an osteochondral autograft procedure with the latter obtaining a success rate of 82%; however, in this patient populations were also ankles included with concomitant tibial lesions (5% in the osteochondral transplantation group and 14% in the repeat arthroscopy group). Since an associated tibial lesion was part of the exclusion criteria and despite multiple requests, it was not possible to obtain the separate data, and therefore, this article had to be excluded. Another remark that has to be made in the light of the study by Yoon et al. [62] is that the authors included talar OCDs with subchondral cysts (up to an incidence of 64% in the repeat arthroscopy group), while in the study by Savva et al. [51] subchondral cysts were deliberately excluded. As stated above, an underlying cyst is associated with inferior outcomes, and this could therefore be a plausible reason for the difference observed in the success rates. Thus, when considering performing a BMS procedure after failed previous surgical treatment, the presence of a cyst should be taken into account when choosing the optimal treatment strategy.

Most of the studies described a more invasive osteochondral or cartilage transplantation method as a subsequent procedure for a previously unsuccessfully treated OCD. In the pooled group, the OATS procedure with an autograft showed the highest success rate. Interesting enough, the pooled success rate of this OATS group which combined 73 treated OCDs resulted in a 90% success rate, which was significantly higher than the 77% success rate in our review about primary-treated OCDs (*p* = 0.0296). The observed difference was a little unexpected and might be explained by differences in size of the treated lesions and concomitant damage to the cartilage of the rest of the ankle joint which were not stated in most studies. Differences in outcome expectancy from the patients can also play a role since the used outcome scores (AOFAS) have a subjective component. Finally, it must be noted that the pooled success rates in the review about primary surgery were all retrospective case series, which were compared to three prospective case series in this review. It is therefore not possible to say whether the difference is due to a clinical or methodological difference.

A difference was found in the autograft versus the allograft procedure with success rates of 90 and 55%, respectively. Differences in these procedures have been previously highlighted in the literature [4, 34, 35, 44]. Since an allograft procedure is typically used for larger defects, it is to be expected that a difference in success percentage was likely to be found. The substantial difference underlines the recommendation that, if possible, an autograft procedure deserves preference over an allograft procedure. The same is the case for the mosaicplasty procedure, in most cases autogenous in nature but also used for larger defects. When comparing the allograft procedure with mosaicplasty, no significant difference was found, and therefore, it is not possible to indicate whether one has superiority in treating larger defects.

One of the major strengths of our systematic review is the contacting with the authors of included studies with the goal of acquiring separate data. This, however, also resulted in a limitation: almost half of the extracted data concerning outcome and success percentages was acquired through the direct approach of the authors which made it virtually impossible to collect all the variables initially being desired to collect in the constructed data set, such as complications reported, lesion size, or classification systems used.

As for pooling of the data, it was not possible to perform a formal meta-regression, that is, utilizing mixed-effects logistic regression in order to compare treatment groups. This is because the number of patients included in the studies was substantially lower than required to obtain stable parameters estimates for this type of analysis [37]. Instead, the authors decided to pool data through a simplified manner where different patients from the same treatment group were added and a new success percentage was calculated. This means that the results presented in the review need to be interpreted with caution. When comparing two different treatment groups, one cannot with certainty state that the difference observed was based on clinical differences or on methodological differences. For example, since the allograft technique is mainly used for the larger defects, it will consequently give a worse outcome.

As for the outcome measurements, the AOFAS score was the most frequently used scoring system. This score as with all the other scores used for success percentage calculation is not officially validated for the clinical evaluation of the treatment of talar OCDs as such. Subsequently, the calculated success percentages have to be interpreted with care. This is clearly exemplified by the outcome reported by the study of Johnson et al. [26]. The adjusted Mazur score was unsatisfactory in the majority of patients, which resulted in a rather low success percentage. However, they found a high average subjective patient-based satisfaction score. This again brings up the question to what extent we should rely on these questionnaires.

The majority of the included studies were of low methodological quality. As long as no randomized *comparative* clinical trials are conducted (such as mosaicplasty versus allograft transplantation or OATS versus AMIC), data will remain insufficient to draw any firm conclusions. These results should therefore not be used in making decisions about technique but rather for prediction of outcome. In clinical practice, this review can be used to illustrate the different treatment techniques and to give patients an indication about the expected success percentages of the different treatment methods for talar OCDs after failed primary surgery.

## Conclusions

In conclusion, multiple diverse surgical treatment options are used for talar OCDs in the case of primary surgical failure. As expected, relatively more invasive methods are administered in comparison with primary treatment. Because of the low level of evidence and the scarce literature reporting on solely non-primary surgery, no methodologically proper meta-analysis could be performed, and it is therefore inappropriate to draw firm conclusions. Besides an expected difference in outcome between the autograft transfer procedure and the more extensive procedures of mosaicplasty and the use of an allograft, neither a clear nor a significant difference between treatment options could be demonstrated. The need for sufficiently powered prospective investigations in a randomized comparative clinical setting remains substantially high.

## Appendix 1

Full electronic search strategy used in this systematic review.

1. PUBMED

**Table:**

#	Searches	Results
1	(“Osteochondritis Dissecans”[Mesh] OR osteochondritis dissecans[tiab] OR osteochondrosis dissecans[tiab] OR osteochondrolysis[tiab] OR ((osteochondral[tiab] OR chondral[tiab] OR transchondral[tiab] OR cartilage*[tiab]) AND (defect*[tiab] OR lesion*[tiab])) OR OCD[tiab] OR OLT[tiab]) AND (“Talus”[Mesh] OR talus[tiab] OR talar*[tiab] OR ankle[tiab])	Total number of results 1996–2016: 986 hits

2. EMBASE (OVID)

**Table:**

#	Searches	Results
1	(osteochondritis dissecans/or (osteochondritis dissecans or osteochondrosis dissecans or osteochondrolysis or OCD or OLT).ti,ab,kw. or ((osteochondral or chondral or osteochondral or transchondral or cartilage*) adj3 (defect* or lesion*)).ti,ab,kw.) and (talus/or (talus or talar* or ankle).ti,ab,kw.)	1351
2	limit 1 to yr = ”1996-Current”	1097

3. COCHRANE LIBRARY

**Table:**

#	Searches	Results
1	MeSH descriptor: [Osteochondritis Dissecans] explode all trees	8
2	osteochondritis dissecans or osteochondrosis dissecans or osteochondrolysis or OCD or OLT:ti,ab,kw (Word variations have been searched)	1117
3	(osteochondral or chondral or transchondral or cartilage*) and (defect* or lesion*):ti,ab,kw (Word variations have been searched)	305
4	#1 or #2 or #3	1410
5	MeSH descriptor: [Talus] explode all trees	27
6	talus or talar* or ankle:ti,ab,kw (Word variations have been searched)	4866
7	#5 or #6	4866
8	#4 and #7, Publication Year from 1996 to 2016, in Cochrane Reviews (Reviews and Protocols), Other Reviews and Trials	23

## Appendix 2

Quality Assessment Scale Utilized NEWCASTLE-OTTAWA QUALITY ASSESSMENT SCALE NONRANDOMIZED STUDIES ADJUSTED FOR CASE SERIES

Note: A study can be awarded a maximum of one star for each numbered item within the Selection and Outcome categories.

*Study Design*

1. Type of Study
  - Prospective*
  - Retrospective
  - Other
  - Not described
2. Set-up
  - According to protocol*
  - Without protocol
  - No protocol described.

*Selection*

3. Representativeness of the exposed cohort
  - Truly representative of the average talar osteochondral defect patient (after failed previous surgery) in the community*
  - Somewhat representative of the average talar osteochondral defect patient (after failed previous surgery) in the community*
  - Selected group of patients by orthopaedic surgeon
  - No description of the derivation of the cohort.

*Outcome*

4. Assessment of outcome
  - Independent blind assessment*
  - Record linkage*
  - Self-report
  - No description.
5. Adequacy of follow-up of series
  - Complete follow-up—all patients accounted for*
  - Subjects lost to follow-up unlikely to introduce bias—small number lost(<5%)*
  - Follow-up rate <95% and no description of those lost
  - No statement.

Number of assigned stars

**Table:**

Study (title, author, year)	Study design	Selection	Outcome

Each included study was graded on methodological quality by two independent reviewers utilizing adjusted version of the Newcastle–Ottawa scale which is included above. Categories of study design, selection of patients, and outcome were scored by means of a scoring system using quantitive amounts of stars, and, respectively, for each category a maximum of 2 stars 1 star, and 2 stars could be obtained (maximum is 5 stars).
